# Synthesis of Potassium Jarosite Solid Solutions: Characterization and Evaluation of Their Potential Electrical Properties

**DOI:** 10.3390/ma19061179

**Published:** 2026-03-17

**Authors:** Felipe Carlos Pérez Olvera, Laura Guadalupe Barajas Martell, Juan Hernández-Ávila, Eduardo Cerecedo Sáenz, Abraham Hernández González, Manuel Saldana, Javier Flores-Badillo, Luis Humberto Mendoza Huizar, Arely M. Gonzalez Gonzalez, Fatima Montserrat Cruz Franco, Estefania Espinosa Morales

**Affiliations:** 1 Academic Area of Earth and Materials Sciences, Universidad Autónoma del Estado de Hidalgo, Pachuca 42184, Hidalgo, Mexico; pe380433@uaeh.edu.mx (F.C.P.O.); ba338551@uaeh.edu.mx (L.G.B.M.); herjuan@uaeh.edu.mx (J.H.-Á.); eduardoc@uaeh.edu.mx (E.C.S.); he397488@uaeh.edu.mx (A.H.G.); javier_flores11060@uaeh.edu.mx (J.F.-B.); cr323332@uaeh.edu.mx (F.M.C.F.); 2 Faculty of Engineering and Architecture, Universidad Arturo Prat, Iquique 1110939, Chile; masaldana@unap.cl; 3 Department of Chemical and Mineral Process Engineering, University of Antofagasta, Antofagasta 1270300, Chile; 4 Academic Area of Chemistry, Universidad Autónoma del Estado de Hidalgo, Carretera Pachuca–Tulancingo Km. 4.5, Mineral de la Reforma 42184, Hidalgo, Mexico; hhuizar@uaeh.edu.mx; 5 Research Laboratory in Nano and Dental Biomaterials, Faculty of Higher Studies Iztacala, National Autonomous University of Mexico (UNAM), Mexico City 54090, Mexico; arely.gonzalez@iztacala.unam.mx

**Keywords:** potassium jarosite, hydrothermal synthesis, X-ray diffraction, voltammetry, Fe^3+^/Fe^2+^ redox processes, ionic conductivity, electrical microgeneration

## Abstract

In this work, the electrochemical behavior of potassium jarosite-type solid solutions synthesized via a controlled hydrothermal method was evaluated. Structural characterization by X-ray diffraction (XRD) confirmed the formation of potassium jarosite. FTIR spectra complemented these findings, revealing bands characteristic of Fe–O metal coordination (625 and 505 cm^−1^). Voltammetric tests evidenced redox processes attributable to the Fe^3+^/Fe^2+^ couple, suggesting that iron within the jarosite framework contributes electrochemically to the observed conductivity. The assembled galvanic cells demonstrated the capability for electrical energy microgeneration, and the presence of jarosite was found to enhance ionic transport within the system. Overall, these results suggest an intergranular ionic-conduction mechanism, possibly facilitated by the mineral matrix, which would act as a structural medium enabling the mobility of charged species.

## 1. Introduction

The growing demand for sustainable alternatives to the intensive use of fossil fuels has driven the development of functional materials for energy-conversion and energy-storage technologies. In this context, the alunite–jarosite supergroup has attracted increasing scientific interest owing to its structural versatility, described by the general formula $\alpha \beta_{3}{\left(\gamma O_{4}\right)}_{2}{\left(\mathrm{OH}\right)}_{6}$, which enables the incorporation of a wide range of cations and anions within its crystal lattice [1]. In these phases, the α site can be occupied by monovalent cations such as K^+^, Na^+^, Ag^+^, or H_3_O^+^; the β site by trivalent metals such as Fe^3+^, Al^3+^, or Cr^3+^; and the γ site by tetrahedral anions such as ${\mathrm{SO}}_{4}^{2-}$, ${\mathrm{PO}}_{4}^{3-}$, or ${\mathrm{AsO}}_{4}^{3-}$ [2]. This compositional flexibility has led to the recognition of more than forty mineral species, among which potassium jarosite, sodium jarosite, and hydronium jarosite are particularly prominent and widely distributed in sulfate-rich natural environments [1].

From a mineralogical perspective, jarosites are characterized by their high iron content, whereas alunites typically exhibit higher proportions of aluminum and sodium in their structure [1]. This distinction reflects specific geochemical conditions—such as pH, dissolved metal-ion concentrations, and the redox potential of the medium—which directly influence the stability and the type of crystalline phase formed [2]. In particular, potassium jarosite (KFe_3_(SO_4_)_2_(OH)_6_) adopts a trigonal structure (space group $R\bar{3}m$), consisting of FeO_6_ octahedra linked to ${\mathrm{SO}}_{4}^{2-}$ tetrahedra and stabilized by interlayer K^+^ cations, which confers notable structural robustness [3]. This phase can form both in natural environments—such as acid mine drainage (AMD) settings—and under synthetic conditions via controlled hydrothermal routes that allow optimization of properties such as phase purity, particle size, and crystallinity [2,4,5].

Owing to these attributes, jarosite has been proposed for environmental and industrial applications, including the immobilization of toxic metals, recovery of valuable metals, and, more recently, the development of electrochemically functional materials [6,7,8]. The latter is particularly relevant in the context of the energy transition, where solid electrolytes are emerging as key components in next-generation electrochemical cells by offering advantages such as enhanced safety, thermal stability, and reduced environmental impact relative to liquid or gel counterparts [4,9]. In this regard, complex sulfate-based materials represent a promising alternative due to their low cost, precursor availability, and ease of synthesis under non-extreme conditions [7].

The mineralogical and geochemical understanding of jarosite has been extensively developed [1,2]. Nevertheless, over the past decade, several studies have addressed its electrochemical properties. Its crystalline framework and its capacity to accommodate diverse cations within the lattice may enable optimized performance in electrochemical energy-related applications. Accordingly, vanadium incorporation into the jarosite structure has been investigated, yielding reversible insertion-type reactions and suggesting a theoretically viable pathway for its use as an electrode material in lithium-ion batteries [10]. Likewise, lead jarosite (Pb-jarosite) has been evaluated as a cathodic component in lithium-ion batteries, exhibiting superior performance compared with sodium jarosite, which has been attributed to its structure and cation vacancies [11]. Along the same line, potassium jarosite has been reported as a pseudocapacitive anode in aqueous lithium-ion hybrid capacitors (ALIHCs), showing good electrochemical efficiency at low temperatures and high capacitance [12].

In another direction, ammonium jarosite has been assessed as a heterogeneous catalyst for the degradation of organic compounds such as polyethylene glycol (PEG) in wastewater, demonstrating favorable catalytic activity [13]. Consistently, ammonium jarosite combined with carbon black has been reported as a catalyst for oxygen evolution during water electrolysis, achieving low overpotentials and reduced Tafel slopes, indicative of strong catalytic performance [14]. It is important to note that jarosite, in its solid and anhydrous state, is not an electrical conductor. However, in the early 2020s, potassium jarosite was evaluated as an anode material, and its electrical properties were reported to be predictable through control of pH, synthesis aging time, and the extent of hydronium incorporation within the structure [2]. Jarosite does not meet the classical criteria of a highly soluble electrolyte; nevertheless, under minimal hydration it can release small amounts of ionic species (K^+^, ${\mathrm{SO}}_{4}^{2-}$, Fe^3+^/Fe^2+^) and sustain constrained ionic transport [15].

These findings highlight the versatility of jarosite, whose composition can be tailored through the incorporation of cations such as Fe^3+^, Al^3+^, K^+^, or ${\mathrm{Na}}^{+}$. Together with functional groups such as OH^−^ and ${\mathrm{SO}}_{4}^{2-}$, such compositional features may facilitate ionic transport under suitable operating conditions. Moreover, controlled synthesis enables adjustment of structural characteristics to optimize electrochemical performance [1,2,5,16]. Therefore, there is a clear need to investigate the potential of jarosite as a functional material in emerging electrochemical systems. Despite its well-known structural stability and abundance of iron-based redox-active species, the literature still reports relatively limited studies focused on its electrical properties and its behavior under oxidation–reduction processes. In this context, the present work provides a novel approach by proposing the electrochemical characterization of an alkaline sulfate of the potassium-jarosite type under controlled conditions. Its response is evaluated by cyclic voltammetry, as well as its performance in Al–Cu and Zn–Cu galvanic cells, with the aim of assessing its properties as a semi-solid electrolyte and its feasibility for energy-conversion applications.

## 2. Materials and Methods

### 2.1. Synthesis of Potassium Jarosite

Potassium jarosite was obtained using a synthesis method based on procedures previously reported in the literature [2,16], which in turn derives from earlier work by other researchers [17]. Under the conditions employed here, this protocol reduces the synthesis time from 24 h to 3 h, lowers the operating temperature from 100^∘^C to 70^∘^C, and maintains controlled pH fluctuations and reagent concentrations. Notably, the present method does not require the addition of auxiliary reagents to reduce particle size.

The synthesis was carried out in 1 L of deionized aqueous solution containing 0.15 mol L^−1^ Fe_2_(SO_4_${)}_{3}\cdot n$H_2_O and 0.15 mol L^−1^ K_2_SO_4_ (purity > 99%, J.T. Baker, Phillipsburg, NJ, USA), which act as precursors for the crystalline framework of potassium jarosite, KFe_3_(SO_4_)_2_(OH)_6_. The reaction was maintained under controlled temperature and stirring conditions for the experimentally established duration. Upon completion, the resulting solid was recovered by filtration and subjected to multiple washes with hot deionized water to remove residual, non-incorporated sulfates. Finally, the product was oven-dried at constant temperature until a homogeneous fine powder was obtained.

### 2.2. Initial Characterization of the Synthesized Material

Structural characterization was performed by X-ray diffraction (XRD) using an INEL Equinox 2000 diffractometer (INEL, Artenay, Centre-Val de Loire, France), with a scan time of 10 min per sample. The diffractograms were processed using MATCH! v3 software (Crystal Impact, Bonn, Germany) for crystalline phase identification. Morphology and semi-quantitative elemental analysis were carried out by scanning electron microscopy (SEM) using a JEOL JSM-IT300 instrument (JEOL Ltd., Tokyo, Japan), located at the Escuela Superior de Apan (UAEH), operated at 30 keV and coupled to an energy-dispersive X-ray spectroscopy (EDS) system.

To verify the incorporation of dopant elements into the structure, Fourier-transform infrared spectroscopy (FTIR) analyses were performed using a PerkinElmer GX59750 spectrophotometer (Waltham, MA, USA) at UAEH. Spectra were acquired over the 500–4000 cm^−1^ range with a resolution of 4 cm^−1^. Samples were prepared by mixing 2 mg of solid with 0.4 g of KBr; the mixture was pressed at 50 kN cm^−2^ for 1 min to form 13 mm-diameter pellets.

### 2.3. Electrochemical Evaluation and Cell Configuration

Electrochemical characterization of the jarosite-type alkaline sulfate was carried out by voltammetry using an EPSILON potentiostat (Bioanalytical Systems, Inc., West Lafayette, IN, USA) controlled with BASi Epsilon EC software (Version 2.00.71_USB). Potential scans were performed from +1.000 V to −1.000 V using a conventional three-electrode configuration, with 0.3 M KCl as the supporting electrolyte. The working electrode was a carbon-paste electrode prepared by mixing 30 wt% of the jarosite-type solid solution with 70 wt% graphite powder. An Ag/AgCl reference electrode with saturated KCl internal solution was used, and a graphite electrode served as the counter electrode.

Electrical conductivity was measured with a portable Oakton PC 700 conductivity meter (range 0.0 μS–200.0 mS; resolution 0.01/0.1/1 μS and 0.01/0.1 mS; accuracy ±1% of full scale). To assess the origin of ionic conduction, conductivity was determined both for the complete suspension (1 g of jarosite in 2 mL of deionized water) and for the supernatant after solid–liquid separation. Measurements were performed at room temperature.

Additionally, the ability of the jarositic material to function as an electrolyte was evaluated. Two galvanic-cell models with the same geometric arrangement were assembled using 30 mL vials, each containing 0.5 g of the jarosite-type solid and 1 mL of deionized water. In both cases, copper (Cu) was used as the cathode, whereas aluminum (Al) and zinc (Zn) were used as anodes, yielding the Al–Cu and Zn–Cu systems, respectively. The cells operated under ambient conditions.

## 3. Results

### 3.1. XRD

**Figure 1:**
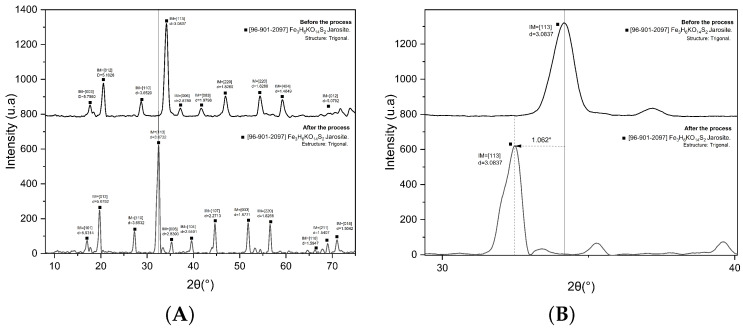
(**A**) X-ray diffractograms of potassium jarosite before and after the discharge treatment. (**B**) Magnified view (zoom) of the most intense peaks, where the shift (displacement) of the diffraction maxima can be observed.

Figure 1A shows the X-ray diffraction (XRD) pattern obtained for the analyzed sample, confirming the predominant presence of potassium jarosite (Fe_3_H_6_KO_14_S_2_). This identification was corroborated by comparison with PDF 96-901-2097. The agreement between the experimental peaks and the reference reflections reported in the database indicates that the jarosite exhibits a trigonal crystal structure (space group $R\bar{3}m$), which is characteristic of this mineral family. Moreover, the presence of sharp, high-intensity peaks suggests a high degree of crystallinity, ruling out the occurrence of any significant amorphous phases.

Figure 1B shows a slight shift in the reflections toward lower 2θ values (≈1.66^∘^), indicating an increase in the interplanar spacing (*d*) according to Bragg’s law. Considering that, under the synthesis conditions and concentrations employed, the effective incorporation of dopant cations into the jarosite-type lattice would be expected to be limited, and given the absence of new reflections supporting the formation of a well-defined solid solution, the observed shift is mainly attributed to effects associated with hydration and/or surface adsorption processes. These phenomena can alter the local environment of structural hydroxyl groups and induce lattice microstrain, which manifests as apparent variations in crystallographic parameters without necessarily implying significant cation substitution at the A site of the structure [18].

**Figure 2:**
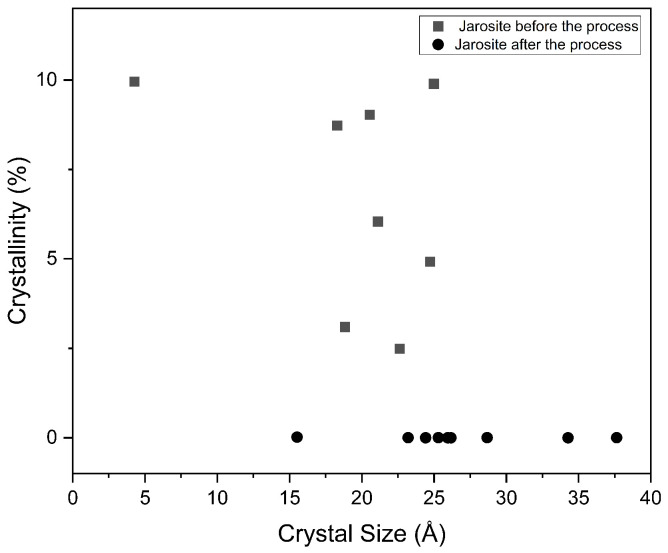
Comparison of particle size and crystallinity percentage in jarosite before and after electrochemical treatment.

These changes in the crystal structure are also reflected in the microstructural properties of the material. As shown in Figure 2, the relationship between crystallite size—estimated using the Scherrer equation—and the degree of crystallinity is presented for potassium jarosite samples before and after the discharge tests. The calculated crystallite size corresponds to the coherent crystalline region at ≈32.42^∘^, consistent with PDF 96-901-2097, where peak broadening is associated with crystallinity according to the corresponding Gaussian or Lorentzian peak shape [19].

For the pre-discharge jarosite sample, a dispersion of crystallite sizes ranging approximately from 5 to 25 Å is observed, accompanied by crystallinity values varying from ∼2% to ∼10%. This distribution suggests the presence of small crystalline domains with a limited degree of structural order. In addition, samples with smaller crystallite sizes tend to exhibit relatively higher apparent crystallinity, which may reflect the contribution of well-defined crystalline regions at the nanometric scale.

In contrast, the post-discharge jarosite sample exhibits larger crystallite sizes, in the approximate range of 15–38 Å; however, the associated crystallinity values are close to zero or significantly low. This behavior indicates that, despite the increase in the average size of coherent domains, the crystalline fraction detectable by XRD is limited. This may be related to a higher degree of structural disorder, an increased density of crystal defects, or the coexistence of a dominant amorphous fraction that does not contribute significantly to coherent diffraction.

### 3.2. Scanning Electron Microscopy (SEM)

**Figure 3:**
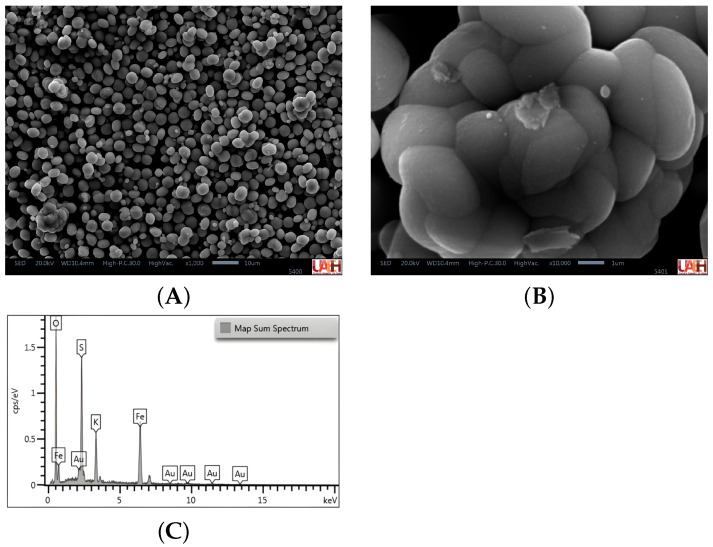
Scanning electron microscopy (SEM) micrographs of jarosite. (**A**) General view showing particle distribution and morphology (magnification 1000×, scale bar 10 µm). (**B**) Detail of an individual particle exhibiting a compact surface and the agglomeration of subunits (magnification 10,000×, scale bar 1 µm). (**C**) Energy dispersive X-ray spectroscopy (EDS) of jarosite.

Figure 3A presents micrographs that reveal the morphological features and the degree of agglomeration of jarosite. Predominantly spheroidal to sub-spheroidal particles are observed, with a relatively homogeneous distribution and average diameters between 1 and 5 μm. These particles coexist with agglomerates formed by the partial coalescence of multiple units. The surfaces are mostly smooth and compact, although some exhibit slight roughness and protrusions, suggesting a controlled nucleation-and-growth process typical of hydrothermal synthesis. This uniformity indicates effective control over particle size and morphology, with potential implications for apparent density, specific surface area, and surface reactivity of the material.

In addition, Figure 3B shows an individual particle with a compact and dense structure, smooth surfaces, and the presence of superficial fractures along with small lamellar incrustations. The morphology suggests hierarchical growth, in which spheroidal grains form through the aggregation of primary subunits. The intimate bonding between these subparticles and the partially vitrified appearance indicate high cohesion and low interparticle porosity. The observed fractures and incrustations may correspond to non-crystallized residues or secondary phases, such as associated sulfates, or to fragments generated during sample preparation.

Complementary to the SEM-based morphological analysis, Figure 3C presents the compositional study by EDS, which enables correlating the observed microstructure with the elemental composition of the material. The EDS spectrum associated with the micrographs confirms the predominant presence of O, Fe, S, and K, which are characteristic elements of the jarosite crystal structure, in agreement with the XRD results. Semi-quantitative analysis showed that oxygen is the major element, followed by iron, sulfur, and potassium, which is consistent with the general jarosite formula ${\mathrm{KFe}}_{3}$(SO_4_)_2_(${\mathrm{OH}}_{6}$). This stoichiometric correspondence supports the successful formation of the target phase and rules out, within the detection limits of the technique, the significant presence of additional metallic impurities.

### 3.3. FTIR

**Figure 4:**
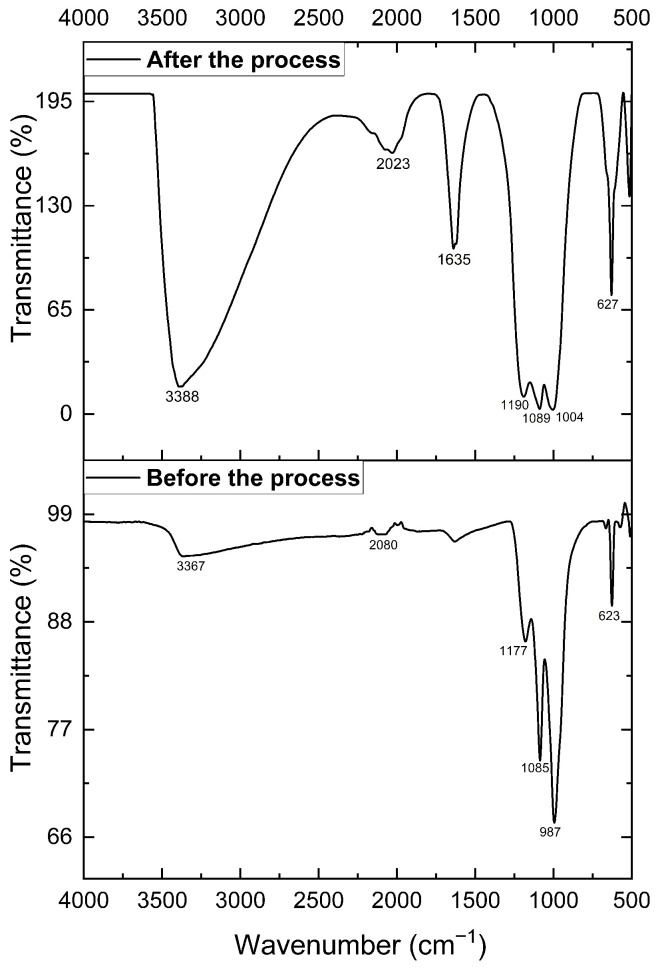
FTIR spectrum of potassium jarosite (JK).

Figure 4 shows the FTIR spectrum of the jarosite-type solid solution, which enables analysis of the atomic vibrational features of the sample. Broad bands centered at approximately 3367 and 3388 ${\mathrm{cm}}^{-1}$ are assigned to ${\mathrm{OH}}^{-}$ groups, corresponding to O–H stretching vibrations of interstitial water and/or metal-bonded hydroxyl groups. In addition, the band observed at 1635 ${\mathrm{cm}}^{-1}$, which can be associated with H_2_O bending modes (H–O–H), appears after jarosite is used in the semi-solid electrochemical cell, suggesting that the sample absorbs water while functioning as an electrolyte [20]. Such water uptake may partially replace monovalent sites within the structure [5,21]. The bands at 1080 and 1190 ${\mathrm{cm}}^{-1}$ are attributed to the asymmetric stretching mode ($\nu_{3}$) of the sulfate anion (${\mathrm{SO}}_{4}^{2-}$). The sharp band in the 987–1004 ${\mathrm{cm}}^{-1}$ region is related to an internal sulfate vibration, most likely the symmetric stretching mode ($\nu_{1}$). Finally, the bands located at 623 and 627 ${\mathrm{cm}}^{-1}$ reflect vibrations associated with Fe–O metal coordination, characteristic of the octahedral ${\mathrm{FeO}}_{6}$ framework [22,23].

### 3.4. Electrochemical Characterization

**Figure 5:**
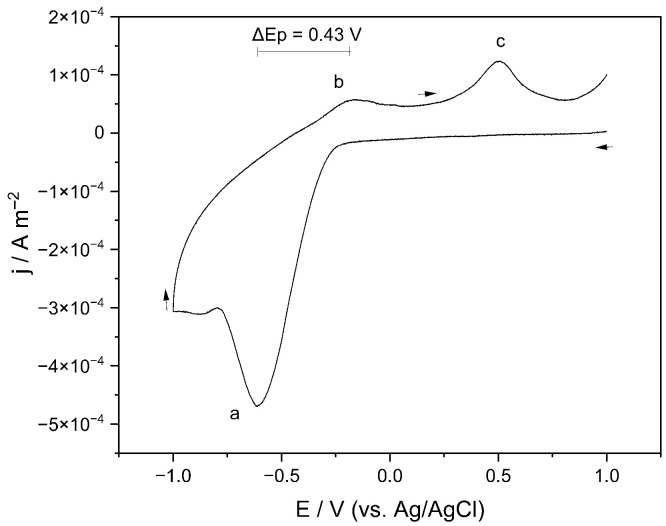
Voltammogram vs. Ag/AgCl of the potassium jarosite-type solid solution in 1 M KCl solution. The potential scan was conducted from +1.000 V to −1.000 V, starting at +1.000 V and proceeding in the cathodic direction. The arrows indicate the scan direction.

Figure 5 shows the voltammogram of the potassium jarosite-type solid solution recorded over a potential window from −1.0 to +1.0 V vs. Ag/AgCl. In the cathodic region, a reduction peak centered at approximately −0.6 V vs. Ag/AgCl is observed, peak a, which can be attributed to the ${\mathrm{Fe}}^{3+}$/${\mathrm{Fe}}^{2+}$ redox couple associated with structural iron within the jarosite crystal lattice. This behavior is consistent with previous studies evidencing the electrochemical participation of structural iron in jarosite-type compounds [22,24]. Unlike systems in which iron is present in solution, in jarosite ${\mathrm{Fe}}^{3+}$ occupies octahedrally coordinated sites within a rigid crystalline framework; therefore, its reduction involves electron injection into structural metal centers. This process may induce local distortions, defect generation, and partial lattice reorganization, as reported for jarosite-type phases under reducing conditions [17,25].

During the anodic sweep, the reverse process is observed at approximately −0.18 V, peak b, corresponding to the re-oxidation of ${\mathrm{Fe}}^{2+}$ to ${\mathrm{Fe}}^{3+}$. However, the separation between the anodic and cathodic peaks ($\Delta E_{p}\approx 0.43$ V) is substantially larger than the theoretical 59 mV expected for a reversible one-electron system at 25^∘^C. This result indicates that the process is not electrochemically reversible but rather under kinetic control. According to heterogeneous electron-transfer theory, large $\Delta E_{p}$ values are characteristic of quasi-reversible or irreversible systems in which electron transfer is limited by interfacial kinetics, the electronic conductivity of the solid, and possible structural reorganizations coupled to the change in oxidation state [26,27]. In crystalline materials with low electronic conductivity, such as jarosites, this behavior is particularly expected.

At more negative potentials (approximately −1.0 V vs. Ag/AgCl), a pronounced increase in cathodic current is observed, which is attributable to faradaic processes other than the structural ${\mathrm{Fe}}^{3+}$/${\mathrm{Fe}}^{2+}$ couple that become activated at the extremes of the potential scan. Similarly, in the more positive anodic region (approximately +0.5 V vs. Ag/AgCl), an additional feature, peak c, is detected that may be associated with surface transformations of the material. Nonetheless, these processes occur outside the main redox region and may correspond to secondary faradaic contributions typically observed in redox-active materials when wide potential windows are applied [28,29].

### 3.5. Evaluation of Electrochemical Performance

To evaluate the ability of the jarosite-type material to act as an ionic medium under minimal-hydration conditions, an experimental prototype was developed based on primary galvanic-cell configurations of two types: Al–Cu and Zn–Cu. A jarosite–water suspension at a ratio of 0.5 g:1 mL was used as the ionic contact phase. Regarding cell geometry, the electrode separation distance was ∼$7\times 10^{-3}$ m, while the effective contact area was ∼$1\times 10^{-5}$ m^2^.

**Figure 6:**
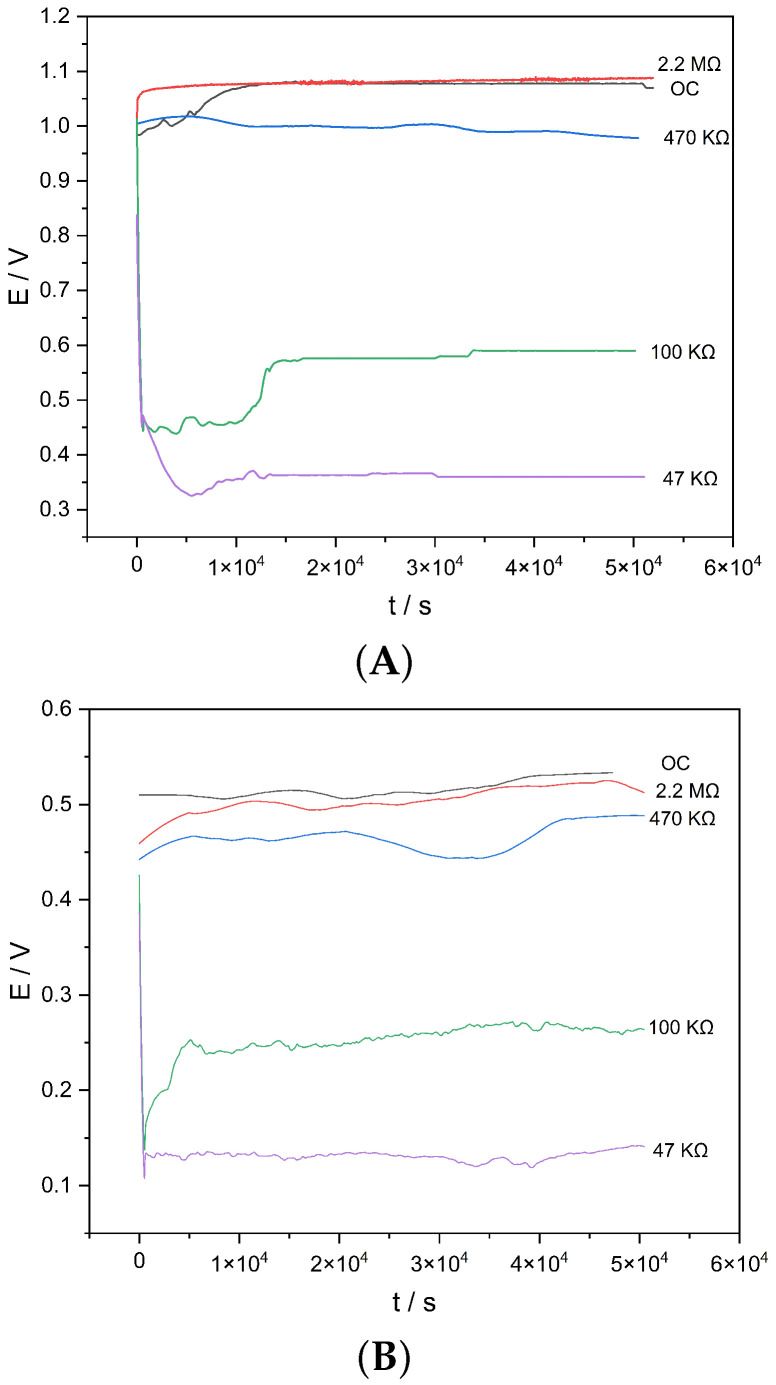
Temporal evolution of the potential (E–t) for (**A**) Zn–Cu and (**B**) Al–Cu cells. The cell contains a jarosite suspension in deionized water (1:2), which acts as the electrolyte. The metallic electrodes were secured to the vial cap using EVA foam and sealed with Parafilm.

The electrical potential was recorded using an Arduino Nano-based acquisition system [30], which enables direct potential measurement while practically eliminating the instrument load impedance, thereby providing a faithful record of the intrinsic potential of the system under different external-resistance conditions. Figure 6 schematically illustrates the cell designed for this experiment, whereas presents the temporal evolution of the potential (*E*–*t*) under the application of different external resistances (2.2 MΩ, 470 kΩ, 100 kΩ, and 47 kΩ), as well as under open-circuit conditions.

For the Zn–Cu configuration, an open-circuit potential of approximately 1.05 ± 0.05 V was recorded, with temporal stability exceeding $5\times 10^{4}$ s. The potential decreased progressively as the external resistance was reduced, reaching values of approximately 0.35 V under 47 kΩ. The response exhibited rapid stabilization followed by a quasi-steady-state regime, indicating the absence of an immediate depletion of mobile species within the partially hydrated matrix. Measurements were limited to the quasi-steady-state regime, since complete cell depletion depends on additional variables associated with the system geometry and the water-evaporation dynamics.

**Figure 7:**
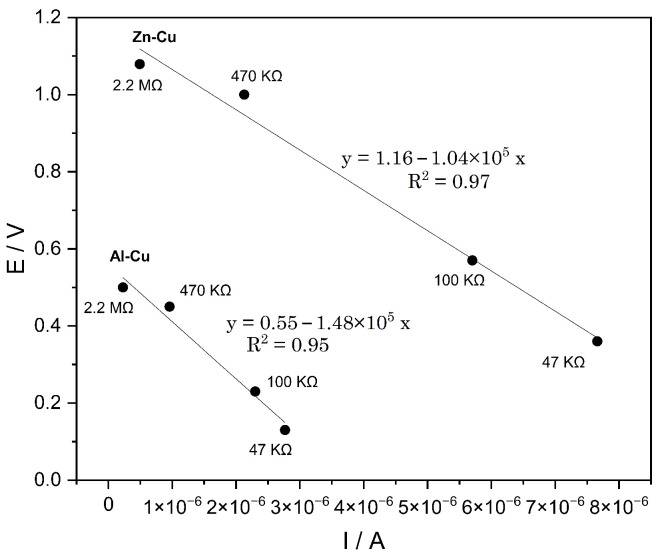
Potential–current (*E*–*I*) dependence derived from the steady-state values under each external resistance for the Zn–Cu and Al–Cu configurations.

In contrast, the Al–Cu configuration displayed an open-circuit potential of approximately 0.50 ± 0.02 V and a larger voltage drop under load, reaching ∼0.12 V at 47 kΩ. More pronounced transient variations were observed during the first seconds of polarization, which can be attributed to interfacial phenomena and to the surface passivation of aluminum. On the other hand, Figure 7 shows the *E*–*I* relationship obtained from the quasi-steady-state values measured under each external resistance.

In both systems, a linear dependence of potential on current was observed, consistent with predominantly ohmic behavior. The intercept on the potential axis corresponds to the extrapolated $E_{\mathrm{oc}}$, whereas the slope represents the effective internal resistance of the system. The obtained values (∼1.0 $\times \;10^{5}$ Ω for Zn–Cu and ∼1.5 $\times \;10^{5}$ Ω for Al–Cu) indicate limited but sustained ionic conduction in the jarosite–water matrix under minimal-hydration conditions. The observed linearity suggests that, within the evaluated current range (microamperes), the system is not dominated by severe diffusional limitations, but rather by an ohmic regime associated with ionic transport through thin water films retained within the mineral structure.

To determine whether ionic conduction originated exclusively from dissolved species or whether the jarosite matrix actively contributed to charge transport, conductivity measurements were performed for both the complete suspension (1 g of jarosite in 2 mL of deionized water) and the supernatant obtained after solid–liquid separation. The conductivity measured for the suspension was 850 μS ${\mathrm{cm}}^{-1}$, whereas the filtered supernatant exhibited a lower value of 732 μS ${\mathrm{cm}}^{-1}$. The ∼16% increase in the presence of the solid phase indicates that ionic transport is not governed solely by ions dissolved in water, but is partially promoted by the jarosite matrix, suggesting a probable intergranular ionic-transport pathway favored by the mineral framework [31].

Complementarily, the effect of the solid phase on the stabilized potential was evaluated in an Al–Cu configuration using a 0.5 g:1 mL ratio. After allowing temporal stabilization of the system, the complete suspension displayed an average potential of 0.502 V, whereas the isolated liquid phase showed 0.470 V. The increase of approximately 32 mV (∼6.8%) in the presence of jarosite confirms that the solid matrix not only enhances the effective conductivity of the medium but also alters the interfacial electrochemical equilibrium.

The metallic foils used weighed 1.16 g for zinc and 0.46 g for aluminum; no appreciable change in mass was observed during the tests, indicating minimal corrosion or material loss over the experimental period. To evaluate the cell arrangement, a preliminary battery was assembled using the same geometry described above. Three cells were connected in series, and the resulting module powered a low-consumption red LED. The potential remained stable up to 1464 h, and then began to decrease gradually; the LED turned off at 1608 h, and the test concluded at 1994 h with a potential of 779 mV, demonstrating the operational lifetime of this battery configuration.

## 4. Discussion

The jarosite synthesized via the hydrothermal method showed a significant match with the reference pattern PDF 96-901-2097, suggesting that its crystal structure and mineralogical composition are equivalent to those of natural jarosite (Figure 1A). This result confirms that hydrothermal synthesis can efficiently reproduce the desired mineral phase and enables adequate control over crystallinity and morphology, thereby overcoming limitations commonly reported for conventional synthesis routes. In addition, Figure 1B evidences a slight shift in the reflections toward lower 2θ values (≈1.66^∘^), which implies an increase in the interplanar spacing (*d*) according to Bragg’s law. Considering that, under the synthesis conditions and concentrations employed, the effective incorporation of dopant cations into the jarosite-type lattice would be expected to be limited, and given the absence of new reflections indicative of a well-defined solid solution, the observed shift is mainly attributed to effects associated with hydration and/or surface adsorption processes. In this regard, the presence of water—whether retained on surfaces, within defects, or in poorly ordered domains—may alter the local environment of structural hydroxyl groups and promote the development of lattice microstrain, leading to apparent variations in crystallographic parameters without necessarily implying significant cation substitution at the A site of the structure [18].

Regarding functionality, potassium-jarosite-type sulfates, even under minimal-hydration conditions, can behave as a matrix capable of retaining water and facilitating ionic transport, albeit with relatively high internal resistances compared with conventional aqueous electrolytes. Nevertheless, the predominantly ohmic response and the temporal stability observed position this material as a candidate for low-power electrochemical systems operating under moisture-restricted scenarios. Finally, the comparative structural analysis of jarosite before discharge (Figure 3) shows that crystallite size and the degree of crystallinity estimated by XRD do not exhibit a direct linear relationship in the evaluated systems. Overall, the results suggest that an increase in the size of coherent diffraction domains does not necessarily translate into a higher global crystalline order, indicating that microstructural features are strongly conditioned by the synthesis route and are reflected in simultaneous variations in crystallite size and crystallinity [18].

Although jarosite does not act as a metallic conductor and its contribution to the system does not arise from electronic transport, it rather stems from intergranular ionic transport facilitated by thin water films retained within the mineral matrix [15,32]. Accordingly, the observed conduction is likely governed by some of the following processes: partial release of K^+^ and ${\mathrm{SO}}_{4}^{2-}$, generation of ${\mathrm{Zn}}^{2+}$ at the anode, and ${\mathrm{Fe}}^{3+}$/${\mathrm{Fe}}^{2+}$ redox reactions. Therefore, the system operates as a controlled-corrosion galvanic cell with an internal resistance on the order of $10^{5}$ Ω. Sustained LED operation for more than two months indicates that the system functions in an ultra-low-power regime, with slow but continuous kinetics, the absence of severe passivation, and partial structural stability of the matrix.

## 5. Conclusions

In this study, potassium jarosite was successfully synthesized via the hydrothermal method, yielding a well-defined and high-purity crystalline phase, as confirmed by X-ray diffraction (XRD), Fourier-transform infrared spectroscopy (FTIR), and scanning electron microscopy (SEM). The results demonstrated that reproducible synthesis under controlled conditions enables the production of a material with homogeneous morphology, high crystallinity, and a particle size suitable for electrochemical applications. The agreement between the observed potentials and standard values reported in the literature further supports the active participation of iron as the predominant electrochemical species in the system.

The results show that jarosite, under minimal-hydration conditions, is capable of sustaining stable ionic conduction over prolonged periods, exhibiting predominantly ohmic behavior with internal resistances on the order of $10^{5}$ Ω. The higher stability and performance observed for the Zn–Cu configuration, together with the increased conductivity measured in the presence of the solid phase relative to the supernatant, confirm that jarosite does not act solely as a passive water reservoir, but rather as a matrix that actively contributes to ionic transport through interfacial mechanisms. Finally, post-discharge structural analyses evidenced significant alterations in the jarosite crystal structure.

Overall, these findings demonstrate the potential of jarosite as an alternative material for constructing wet solid-matrix batteries, particularly in systems characterized by low ionic mobility. Its use is especially promising for electrochemical microgeneration devices and ultra-low-power equipment. Although such systems are not intended to compete with conventional commercial batteries in terms of energy density or power output, they represent a viable alternative for long-life autonomous devices with minimal energy requirements. Moreover, they could be deployed in extreme or hard-to-access environments, where the availability of natural minerals would facilitate implementation and reduce the need for frequent maintenance or replacement. In this context, jarosite should be regarded not as a direct substitute for current commercial technologies, but as a complementary solution aimed at specific applications in which stability, material availability, and ultra-low energy demand are determining factors.

## Data Availability

The original contributions presented in this study are included in the article. Further inquiries can be directed to the corresponding author.

## Abbreviations

The following abbreviations are used in this manuscript:

XRD	X-ray diffraction
FTIR	Fourier transform infrared spectroscopy
SEM	Scanning electron microscopy
